# Are the Norwegian health research investments in line with the disease burden?

**DOI:** 10.1186/1478-4505-12-64

**Published:** 2014-11-27

**Authors:** Jonas Minet Kinge, Ingrid Roxrud, Stein Emil Vollset, Vegard Skirbekk, John-Arne Røttingen

**Affiliations:** Norwegian Institute of Public Health, Postboks 4404, Nydalen, 0403 Oslo, Norway; The Research Council of Norway, P.O Box 564, 1327 Lysaker, Norway; Department of Global Public Health and Primary Care, University of Bergen, Postboks 7800, 5020 Bergen, Norway; Columbia Aging Center, Columbia University, 722 W. 168th Street, New York, NY 10032 USA; Department of Health Management and Health Economics, University of Oslo, P.O box 1130, 0318 Oslo, Norway

**Keywords:** Disability adjusted life years, Global health, Health research, Norway, Research funding, Years of life lost

## Abstract

**Background:**

The relationship between research funding across therapeutic areas and the burden of disease in Norway has not been investigated. Further, few studies have looked at the association between national research investments and the global disease burden. The aim of the present study was to analyze the correlation between a significant part of Norwegian investment in health research and the burden of disease across therapeutic areas, using both Norwegian and global burden of disease estimates.

**Methods:**

We used research investment records for 2012 from the Research Council of Norway, and the investment records distributed through liaison committees between regional health authorities and universities. Both were classified by the Health Research Classification System (HRCS). Furthermore, we used the years of life lost and Disability Adjusted Life Years (DALYs) for Norway and globally from the Global Burden of Disease 2010 project. We created a matrix to match the expenditures by HRCS with the values from the Global Burden of Disease project.

**Results:**

Disease-specific research funding increased with the Norwegian burden of disease measured as years of life lost (correlation coefficient = 0.73). Similar findings were done when the Norwegian disease burden was measured as DALYs (correlation coefficient = 0.62). The correlation between research funding and the global disease burden was low both when years of life lost (correlation coefficient = 0.11) and DALYs (correlation coefficient = 0.12) were used. Generally, when the disease burden was relatively high in Norway compared with the rest of the world, research investments were also high.

**Conclusions:**

Across therapeutic areas, the Norwegian research investments appeared aligned with the Norwegian disease burden. The correlation between the Norwegian research investments and the global disease burden was much lower.

**Electronic supplementary material:**

The online version of this article (doi:10.1186/1478-4505-12-64) contains supplementary material, which is available to authorized users.

## Background

In the US in 1997 the National Institute of Health’s working group on priority setting recommended using public health needs as a criterion for research [1]. Similar recommendations have later been made in other countries [2], which has motivated research to assess the correlation between research funding and measures of disease burden. Some of these studies, which have been conducted in the US, the UK, and Australia, reveal high correlations between research funding and measures of disease burden [1, 3–7]. However, other studies dispute this [2, 6, 8, 9], and cancer, in particular, appears to attract more funds relative to disease burden compared with other diseases [2, 5, 7, 9]. Some of the literature in this field has examined different cancers with the general finding that breast cancer attracts more funding relative to disease burden than other types of cancer [1, 3, 6, 8].

While the association between research funding and burden of disease has been investigated in several countries, little attention has been paid to national research investments and global disease burden. Such considerations are important given recent calls for new strategies for incorporating a global perspective into investments in health research [10–12]. Health threats, as a result of globalization, are becoming increasingly transnational and social determinants of health at the national level are influenced by global markets, migrations, and communication. Efficient management of global health resources are thus needed. Furthermore, it can be argued that Norway, as a rich country, has a special responsibility to conduct research that reflects the health needs of developing countries [13].

It has been suggested that health need is a key criterion for deciding the allocation of resources for both treatment and research [14, 15]. However, health need alone is not a sufficient criterion to determine research priority, i.e., twice the health need does not justify twice the investment in research. Such a decision must also depend on the opportunity for progress in the area. Without this consideration, less overall scientific progress in the field of health research would be accomplished, which would lead to less of a reduction in the burden of disease than what could have been achieved otherwise. In addition, there are a range of other criteria, such as feasibility of the intervention, equity, and cost-effectiveness, which must be included in a comprehensive analysis to ensure that important considerations are not overlooked. Hence, this is not a prioritization analysis, but rather a contribution to the debate on global research funding.

The aim of the following analysis was to assess the correlation between Norwegian research investments and both the Norwegian and global burden of disease. This was possible as both the Research Council of Norway and the Regional Health Authorities have adopted the International Health Research Classification System (HRCS) to classify part of their efforts in health research [16]. As a measure of Norwegian and global morbidity/mortality, we used data from the International Global Burden of Disease (GBD) 2010 project [17, 18].

### Data and methods

#### Research funding

Research in health and medical sciences performed by public institutions in Norway is financed through different channels, including the Ministry of Health and Care Services, the Ministry of Education and Research, the Research Council of Norway (RCN), the Regional Health Authorities (RHA), charities, international sources, and private companies [19]. Charities, international sources, and private companies, however, were only responsible for 12% of the total investments in public sector health research in 2011; thus, 88% was publicly funded [20]. In comparison with other high income nations, Norway has a low involvement of the private sector in health research [21].

We used portfolio data from the RCN [22] and RHAs in 2012 to calculate research activity and investments. Resource allocation was calculated based on the financial investments in each of the projects in 2012. The RCN has used the HRCS since 2011 and we included, from the RCN, investments related to the target area “Better health and health care”. This amounted to NOK 783 million (EUR 104 million), which was approximately 85% of the RCN’s overall investments in the health field in 2012.

The RHAs have, since 2009, used HRCS to classify a part of their projects. Hence, we could include approximately 29% (NOK 810 million, EUR 107 million) of the RHA’s total expenditures (NOK 2.8 billion, EUR 371 million) on research in 2012 [23]. We included the part of their portfolios which was funded through liaison committees between the RHAs and the universities. Most of these funds are earmarked research allocations from the Ministry of Health and Care Services to the specialist health care services. However, some are also basic funding from block grants to a broadly defined type of activity, including patient treatment, education, and research. The part that is not classified by HRCS (NOK 2 billion, EUR 264 million), and thus not included in our analysis, consists mostly of block grants to broadly defined activities. It is not known if the HRCS profiles we observe are representative of the overall research effort in the RHAs [24].

In total, we include an amount of roughly NOK 1.6 billion (EUR 211 million) in research funding. The total research investments in the public sector in the field of medicine and health have not been estimated for 2012. However, in 2011 this was NOK 6.2 billion (EUR 822 million) [20].

#### Measures of burden of disease

Several health-based burden measures have been proposed (e.g., disability-adjusted life years (DALYs), quality-adjusted life years, years of life lost (YLLs), and mortality), which have different characteristics. In the following study, we apply YLLs and DALYs, for Norway and globally, from the GBD 2010 project [17, 18]. GBD 2010 is an international project based at the Institute for Health Metrics and Evaluation at the University of Washington. The aim of the project is to provide a comparative overview of population health and mortality as well as associated risk factors. The results are stratified by age, gender, region, and country. YLL measures loss of life due to premature mortality and is calculated by subtracting the age at death from the life expectancy for a person at that age. The use of DALYs was initiated by the World Bank and WHO as an overall measure of disease burden [25]. DALYs equal the sum of YLLs and years lived with disability. Hence, this measure incorporates loss of life due to nonfatal health conditions with one DALY equal to the loss of one year of healthy life.

The disease groups used in the HRCS and GBD categories are not directly comparable. Hence, we created our own disease groups, which are displayed (Additional file 1). An important weakness is that we could not match the HRCS categories “Other” and “Generic Health Relevance” with the disease-specific categories of the GBD. These accounted for 30% of the RCN’s investments and 13% of the RHAs’ investments, thus constituting 22% of the total investments in the analysis.

To illustrate the association between research investments and disease burden, we used scatter plots supplemented with linear trends. We also calculated correlation coefficients (Pearson’s) for each association.

## Results

The share of the total research investment across the disease categories ranged from 19.4% in cancer to 0.5% in injuries (Table 1). The disease category responsible for most YLLs in Norway was cancer, followed by cardiovascular disease. Musculoskeletal disorders, mental health, and cancer caused most DALYs in Norway. Infections were most important both in terms of YLLs and DALYs at the global level. Cardiovascular disease was the second most important reason for both YLLs and DALYs globally. The two last columns in Table 1 show the relative ratio of Norwegian YLLs and DALYs compared to global YLLs and DALYs. When the ratio is above 1, the relative disease burden in Norway was higher than the global burden; conversely, ratios below 1 show that the relative disease burden globally was higher than the Norwegian burden. In terms of YLLs, mental health was 3.26 times as important for the total burden of disease in Norway compared with globally. Musculoskeletal disorders were 2.6 times more important for the total burden of disease in Norway compared with the global burden, in terms of DALYs lost. On the other hand, the burden from “reproductive health and childbirth” and infectious diseases was much higher globally in terms of both YLL and DALYs.

**Table 1:** **Research investments in Norway in 2012 and the Norwegian and global burden of disease, measured by years of life lost (YLLs) and disability-adjusted life years (DALYs)**

	Percentage share of total research investments			Norway		Global		Rates	
Disease categories	RCN, NOK 783 mill.	RHAs, NOK 810 mill.	RCN + RHAs, NOK 1,593 mill.	% of YLLs	% of DALYs	% of YLLs	% of DALYs	Norway YLLs/Global YLLs	Norway DALYs/Global DALYs
Musculoskeletal disorders	2.0%	4.3%	3.2%	0.6%	17.0%	0.2%	6.5%	2.00	2.6
Cancer	15.5%	23.1%	19.4%	32.8%	15.5%	10.7%	7.6%	1.78	2.0
Mental health	12.0%	12.8%	12.4%	3.8%	15.6%	0.5%	7.4%	3.26	2.1
Neurological disorders	10.3%	8.3%	9.3%	5.2%	5.5%	1.8%	3.0%	2.78	1.8
Metabolic^a^	3.1%	4.1%	3.6%	2.6%	3.8%	2.0%	2.3%	0.86	1.6
Cardiovascular diseases	7.1%	13.0%	10.1%	27.9%	14.4%	15.9%	11.5%	1.27	1.3
Blood/urogenital^b^	0.4%	2.55%	1.48%	1.6%	2.70%	1.9%	2.52%	1.06	1.1
Respiratory diseases	1.4%	0.9%	1.2%	5.3%	5.0%	4.0%	4.8%	0.89	1.1
Other non^c^	0.8%	1.8%	1.3%	1.5%	5.1%	2.4%	5.1%	0.43	1.0
Oral and gastrointestinal^d^	1.2%	2.0%	1.6%	3.1%	1.8%	3.4%	2.6%	0.81	0.7
Injuries	0.6%	0.4%	0.5%	10.0%	8.9%	13.5%	11.1%	0.59	0.8
Reproductive health^e^	2.9%	3.5%	3.2%	1.6%	0.9%	12.2%	8.9%	0.05	0.1
Infection	7.6%	2.6%	5.0%	4.2%	2.8%	29.6%	22.8%	0.33	0.1
Correlation with the RCN investments				0.59	0.51	0.19	0.24	0.63	0.41
Correlation with the RHAs investments				0.80	0.67	0.05	0.04	0.57	0.53
Correlation with total investments (RCN + RHAs)				0.73	0.62	0.11	0.12	0.61	0.49

RCN, Research Council of Norway; RHA, Regional Health Authorities.

^a^And endocrine diseases.

^b^Diseases.

^c^Communicable disorders.

^d^Diseases.

^e^And childbirth.

The correlation coefficients demonstrate a high correlation between the total research investments included in the analysis and associated disease burden, in terms of YLLs and DALYs, in Norway (Table 1). The correlations between the Norwegian disease burden and total research investment were 0.73 for YLLs and 0.62 for DALYs. The correlations between total research investments and the global disease burden were 0.11 for YLLs and 0.12 for DALYs and were thus considerably weaker than in the Norwegian case. The correlation between Norwegian disease burden and investments was more pronounced for the RHAs than for the RCN. In contrast, at the global level, RCN investments correlated more with disease burden than the RHAs’ investments. The correlations between the RHAs’ investments and global YLLs and DALYs were 0.05 and 0.04, respectively, i.e., almost non-existent.

The share of total research investments increased with the relative ratios. This means that disorders with a particularly high disease burden in Norway, relative to the global burden, attracted relatively more investments. Conversely, it could mean that less was invested if the disease burden was low in Norway relative to the rest of the world. The positive correlation was higher for RHAs (0.53) than for the RCN (0.41). This suggests that RHAs are more focused on the Norwegian disease burden, while the RCN has a more global profile.

**Figure 1:**
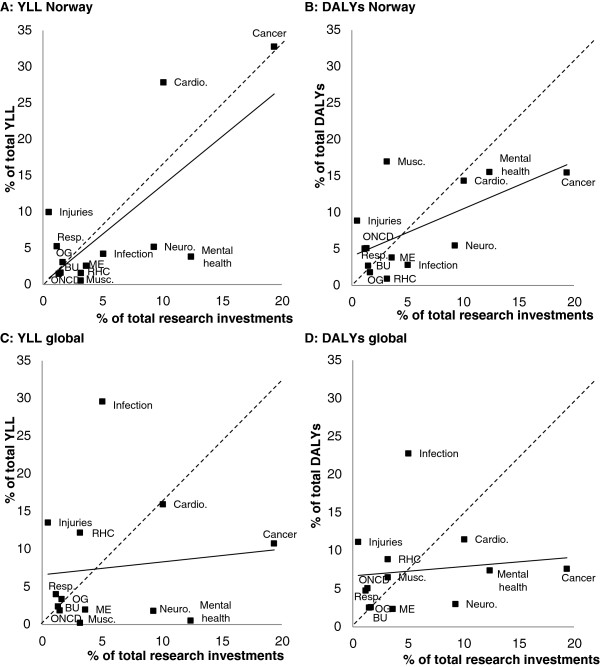
**Scatter plots with research investments and the Norwegian YLLs (A), Norwegian DALYs (B), global YLLs (C), and global DALYs (D).** Note: the solid line lines depicted in each figure are the best-fit linear regression lines and the dashed lines show where funding match the burden. BU, Blood and Urogenital disorders; Cardio, Cardiovascular diseases; ME, Metabolic and endocrine disorders; Musc, Musculoskeletal disorders; Neuro, Neurological disorders; OG, Oral and Gastrointestinal disorders; ONCD, Other non-communicable diseases; Resp, Respiratory diseases; RHC, reproductive health and childbirth.

Figure 1A–D displays the results from Table 1 with scatter plots supplemented with linear regression lines of the correlations. Figure 1A illustrates how the disease category cancer, which accounted for a large proportion of the YLLs, had the highest research investments. We also see that cardiovascular diseases received less funding than both cancer and mental health. Figure 1B demonstrates the consequence of taking both YLLs and morbidity into account through DALYs. Compared with YLLs, both cardiovascular disease and cancer are relatively less important, while the magnitude of health loss from both musculoskeletal diseases and mental health increases significantly.

Figure 1C, D illustrates the results based on the global YLLs and DALYs. Although the global trends were similar to those of Norway, there are important differences of which the most important is the increased burden from infectious diseases. As Norway funds relatively little research on this disease category, the correlations are weaker than that seen when comparing with the Norwegian disease burden.

**Figure 2:**
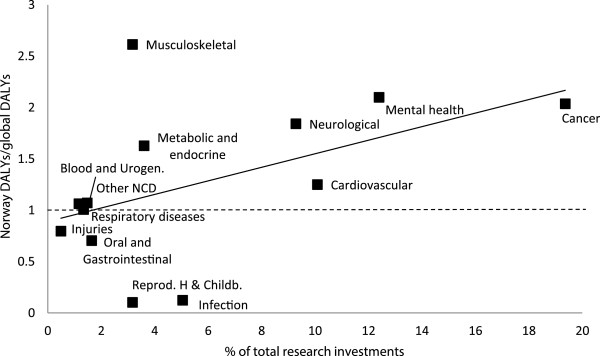
**Scatter plot with the association between total research investments and rates (% Norway DALYs/global DALYs).** The solid line depicted is the best-fit linear regression line. Blood and Urogen, Blood and Urogenital; Reprod H & Childb, Reproductive health and childbirth.

Figure 2 illustrates the relative Norwegian DALYs to the global DALYs by disease category. This is mapped against the total research investment included in the analysis. As shown in the figure, the burden of musculoskeletal disorders has been especially high in Norway, but the research investments have been relatively low. The burden of diseases related to reproductive health and childbirth as well as infectious diseases was very small in Norway. With the exception of musculoskeletal disorders, the RCN and RHAs invested more in diseases in which the disease burden was high in Norway compared with the rest of the world.

## Discussion

We found that a significant part of research investment in the health sector in Norway was highly correlated with the disease burden in Norway, although with some exceptions. This is important as it demonstrates that investments in research largely follow the health needs of the Norwegian population. The association between investments and the Norwegian disease burden was more pronounced for the RHAs compared with the RCN. In addition, we compared the Norwegian research investments with the global disease burden and found a positive correlation. However, the association was less pronounced, compared to the correlation between research investments and the Norwegian disease burden. Finally, we illustrated, using rates, that when the relative burden in Norway was high, compared with the global burden, this disease attracted more funds.

Different measures of disease burden can be used to focus attention on different aspects of the disease [26, 27]. YLLs measures have been found to better predict research funding than mortality [1, 27]. However, composite measures of disease burden that include both morbidity and mortality, like DALYs, have been found to correlate more with funding that YLLs [1, 3]. We explored the use of two measures of disease burden – YLLs and DALYs – and found that the choice of either measure highly influenced the ranking of disease categories. This disparity was primarily manifested within non-fatal disorders. For example, musculoskeletal and mental health disorders had a low impact on disease burden when YLLs was used as the sole criterion. However, when a composite measure of both mortality and morbidity through DALYs was applied, the burden from such disorders increased vastly. Such variations demonstrate how policy makers could be misled. By using a single measure of burden, advocates interested in promoting research on specific diseases could select measures that support their cause [1].

Norway, as a rich country, may have a special responsibility to conduct research that reflects international priorities and contribute knowledge that can support the formulation of international health initiatives. Further, the disease burden is a central criterion for setting priorities in health research [14, 15, 28]. Our analysis shows that musculoskeletal, neurological and mental health illnesses were less important for the global burden compared with the Norwegian burden. Conversely, infectious diseases, reproductive health, and childbirth complications were pivotal globally, particularly in developing countries. One might argue that these diseases deserve particular attention as developing countries with low GDP per capita have few resources available for health research [15].

The findings suggest that the RCN investments are more globally oriented than those of the RHAs. For example, a larger share of the RCN’s investments was invested in research on reproductive health and childbirth complications, while the RHAs invested more in musculoskeletal disorders. This is not surprising given that the RHAs are responsible for covering all clinical areas and invest in clinical research to a greater extent than the RCN. In addition, hospitals indeed pay particular attention to local needs, while the RCN has a major program initiative in global health research.

In the portfolios we have analyzed, only a minor part were the direct result of overall prioritization of certain disease areas over others. It is therefore interesting that, in a collection of portfolios which are distributed mainly based on competition and without prioritized disease areas, we see a positive correlation between financial input and disease burden. This is a sign that the Norwegian research system seems to sufficiently cover the range of clinical activity and that there is room for high quality research on most disease areas of importance.

We emphasize that the need criterion is not sufficient for an efficient allocation of resources and cannot be used exclusively for priority purposes as important parts are lacking. The goal of activities to set priorities for health research is to define an investment portfolio of health research and development that will have the greatest possible impact on the health of the majority of the population [14]. To achieve this, other aspects must be taken into the analysis such as the efficiency criterion (the higher the priority, the higher the potential health benefits) and the cost-effectiveness criterion (the higher the priority, the greater the health benefit per invested funds). To conduct a full prioritization analysis one would need additional evidence on the costs of the disorders and, importantly, the potential for scientific progress [1]. Further, there are other important goals and drivers in Norwegian research policy such as the need to stimulate a more research-intensive private sector, foster high quality research and scientific excellence, and utilize national advantages. Different criteria are applied depending on the context and research area.

### Limitations

Our study has several limitations. First, our analysis is limited by data availability as we have only been able to classify parts of the total research investments in the public sector in Norway. For example, we have not classified the basic funding to institutions such as universities and university hospitals, nor the funding stemming from charities and private investments. Without taking these types of funding into account, there is some uncertainty regarding the generalizability of our therapeutic specific classification as such institutions may prioritize funding differently than those under consideration in this study. Although private investments and charities only accounted for 12% of the total investments in the public sector in Norway in 2011, studies have found that they invest differently than governmental agencies [2]. For example, in the UK, they found that charities invest relatively more in cancer research [2].

Second, the burden of disease may be a result of investments in research, i.e., a low disease burden in some categories might be a result of the Norwegian, and perhaps above all the global, health research community investments into research on these diseases.

Third, our study hides the importance of general health service research. There may be a burden from diseases where effective prevention and treatment already exists, yet resource constraints and local expertise limits utilization and access. Hence, knowledge from research on a specific clinical topic may be sufficient, but there is a need for more knowledge about how health services can be delivered and reach those with the greatest need.

Fourth, when using composite measures of disease burden, which includes both the quality and quantity of life lived, one must take into account that they reflect the disability weight given to each health state. The use of DALYs, as calculated by the GBD, to represent health need is controversial for a number of reasons. DALYs have conceptually been criticized for not reflecting health need. The elicitation methods used to obtain the values for non-fatal health conditions may have consequently caused low disability weights for certain physical disabilities [29]. A number of disabilities, such as blindness and deafness for example, are not directly perceived as a health loss. As a result, severe and chronic neck pain is ten times worse than complete hearing loss [29] and this affects the ranking of the health states. In addition, it is important to recognize that the estimation of the true health loss from a disability may vary by region. Wealthier regions may have a different health loss from similar disorders compared with poorer regions [8].

## Conclusions

The aim of the current study was not to suggest how much Norway should spend on particular disease groups, but rather to inform decisions on the distribution of research effort. The research investments included in the analysis were highly correlated with the Norwegian disease burden. However, the correlation with the global disease burden was much lower. Generally, diseases that were relatively important in Norway compared with the rest of the world attracted more investments.

## Electronic supplementary material

Additional file 1:**The composition of our disease categories.**(DOCX 20 KB)

## Abbreviations

DALY: Disability adjusted life years
GBD: Global Burden of Disease
HRCS: Health Research Classification System
RCN: Research Council of Norway
RHA: Regional Health Authorities
YLL: Years of life lost.
