# Supplementary material for: Are the Norwegian health research investments in line with the disease burden?
Source: Health Res Policy Syst. 2014 Nov 27;12:64. doi: 10.1186/1478-4505-12-64 (PMC4256793; doi:10.1186/1478-4505-12-64)
Supplement: Supplementary file 1 — Additional file 1:The composition of our disease categories.(DOCX 20 KB) [file 12961_2014_361_MOESM1_ESM.docx]

**Additional file 1** the composition of our disease categories

| ***Our categories*** | **GBD categories** |  | **HRCS categories** |
| --- | --- | --- | --- |
| *Cancer* | - Cancer |  | - Cancer |
| *Injuries* | - Intentional injuries - Unintentional injuries - Transport injuries |  | - Injuries and Accidents |
| *Musculoskeletal disorders* | - Musculoskeletal disorders |  | - Musculoskeletal |
| *Mental health* | - Mental & behavioural disorders |  | - Mental Health |
| *Neurological disorders* | - Neurological disorders |  | - Neurological |
| *Reproductive Health and Childbirth* | - Neonatal disorders - Maternal disorders - SIDS |  | - Reproductive Health and Childbirth |
| *Infection* | - Diarrhea/LRI/other infectious - HIV/AIDS & tuberculosis - NTD & malaria - Other communicable |  | - Infection |
| *Cardiovascular diseases* | - Cardio & circulatory diseases |  | - Cardiovascular - Stroke |
| *Respiratory diseases* | - Chronic respiratory diseases |  | - Respiratory |
| *Oral and Gastrointestinal diseases* | - Digestive diseases - Cirrhosis |  | - Oral and Gastrointestinal |
| *Other non-communicable disorders* | - Other non-communicable |  | - Skin - Ear - Eye - Congenital Disorders |
| *Metabolic and endocrine diseases* | - Diabetes - Other endocrine |  | - Metabolic and Endocrine |
| *Blood/Urogenital diseases* | - Hemoglobinopathies - Urinary diseases - Chronic kidney disease - Glomerulonephritis - Gynecological diseases |  | - Blood - Renal and Urogenital |
